# Isolated IgG2 deficiency is an independent risk factor for exacerbations in bronchiectasis

**DOI:** 10.1093/qjmed/hcab129

**Published:** 2021-05-10

**Authors:** Y Zhang, A Clarke, K H Regan, K Campbell, S Donaldson, J Crowe, A G Rossi, A T Hill

**Affiliations:** The Centre for Inflammation Research at the University of Edinburgh, Queen’s Medical Research Institute, Edinburgh BioQuarter, Edinburgh EH16 4TJ, UK; The Centre for Inflammation Research at the University of Edinburgh, Queen’s Medical Research Institute, Edinburgh BioQuarter, Edinburgh EH16 4TJ, UK; Department of Respiratory Medicine, Royal Infirmary of Edinburgh, Edinburgh EH16 4SA, UK; The Centre for Inflammation Research at the University of Edinburgh, Queen’s Medical Research Institute, Edinburgh BioQuarter, Edinburgh EH16 4TJ, UK; Department of Respiratory Medicine, Royal Infirmary of Edinburgh, Edinburgh EH16 4SA, UK; Department of Respiratory Medicine, Royal Infirmary of Edinburgh, Edinburgh EH16 4SA, UK; The Centre for Inflammation Research at the University of Edinburgh, Queen’s Medical Research Institute, Edinburgh BioQuarter, Edinburgh EH16 4TJ, UK; Department of Respiratory Medicine, Royal Infirmary of Edinburgh, Edinburgh EH16 4SA, UK; Department of Respiratory Medicine, Royal Infirmary of Edinburgh, Edinburgh EH16 4SA, UK; The Centre for Inflammation Research at the University of Edinburgh, Queen’s Medical Research Institute, Edinburgh BioQuarter, Edinburgh EH16 4TJ, UK; The Centre for Inflammation Research at the University of Edinburgh, Queen’s Medical Research Institute, Edinburgh BioQuarter, Edinburgh EH16 4TJ, UK; Department of Respiratory Medicine, Royal Infirmary of Edinburgh, Edinburgh EH16 4SA, UK

## Abstract

**Background:**

Immunoglobulin G (IgG) subclass 2 deficiency is the most frequent IgG subclass deficiency identified in patients with bronchiectasis, but its clinical significance is not known.

**Aim:**

To analyse if bronchiectasis patients with isolated IgG2 deficiency at risk of recurrent exacerbations and/or hospitalization? Do patients with IgG2 deficiency have worse disease progression?

**Design and Methods:**

This is a retrospective study (2015–20) exploring independent risk factors for recurrent exacerbations (3 or more per year) and/or hospitalization with bronchiectasis exacerbations using multivariable models using binary logistic regression. There was no patient with IgG deficiency, IgG 1, 3 or 4 deficiency, or IgA or IgM deficiency included. In this model, the authors included: serum IgG2 level; lung function; body mass index; MRC breathlessness scale; age; sex; number of bronchiectatic lobes; bacterial colonization; comorbidities; and the use of long-term immunosuppressant drugs or antibiotics for more than 28 days. Analysing 2-year longitudinal data, one-way ANOVA and Mann–Whitney U-test were used to compare bronchiectasis severity between patients with different IgG2 levels.

**Results:**

Serum IgG2 levels (<2.68 g/l, 2.68–3.53 g/l and 3.54–4.45 g/l); hospital admission in the preceding 2 years; bacterial colonization with potentially pathogenic organisms and asthma were independent predictors for three or more bronchiectasis exacerbations. Those with low IgG2 levels (<2.68 g/l and 2.68–3.53 g/l), had worsening progression of their bronchiectasis, using the Bronchiectasis Severity Index, over 1 year compared with those who were IgG2 replete (>4.45 g/l) (*P* = 0.003, 0.013).

**Conclusion:**

Reduced IgG2 levels were an independent predictor for bronchiectasis exacerbations and have increased disease progression.

## Introduction

Patients with bronchiectasis are at risk of exacerbations, the average number of exacerbations is ∼2.6 per patient per year, 18–38% patients had 3 or more exacerbations every year.^1–4^ Patients having 3 or more bronchiectasis exacerbations per year have significantly higher rates of hospitalization and mortality.^5^ British Thoracic Society guidelines recommend consideration of long-term antibiotics for those patients.^6^

Immunoglobulin G (IgG) is the major serum immunoglobulin.^7^ It can recognize, neutralize and eliminate pathogens and toxic antigens. IgG deficiency leads to increased frequency and severity of mucosal and systemic infections, especially respiratory infections.^8^^,^^9^

IgG are divided into four subclasses: IgG1, IgG2, IgG3 and IgG4.^8^^,^^10^ IgG and IgG subclass deficiencies are the most common (66.5%) primary immunodeficiencies in children with bronchiectasis,^11^ with IgG2 deficiency being the most common (16–29%) IgG subclass deficiency associated with bronchiectasis.^12^^,^^13^ IgG2 is particularly important in the pulmonary immune response against bacteria, as it mediates the phagocytosis of encapsulated bacteria.^14^ Bronchiectasis patients with IgG2 deficiency exhibit impaired antibody responses to *Haemophilus B* conjugate vaccines and pneumococcal vaccines.^15^^,^^16^ In chronic obstructive pulmonary disease (COPD), IgG deficiency leads to 50–100% higher rate of both exacerbations and hospitalizations and IgG2 deficiency is an independent predictor for both.^17^ The clinical significance of IgG subclass 2 deficiency in bronchiectasis is not well understood and merits further study.^18^

The aim of this study was to assess whether isolated IgG2 deficiency are at risk of recurrent exacerbations (three or more per year) and/or hospitalization for bronchiectasis? Do patients with isolated IgG2 deficiency have worse disease progression?

## Study design and methods

This is a retrospective study (2015–20) exploring independent risk factors for the number of exacerbations per year and/or hospitalization with bronchiectasis exacerbations using multivariable models using binary logistic regression.

### Patient inclusion criteria

Patient data and serum samples analysed in this study were obtained from patients attending the bronchiectasis clinic, Royal infirmary of Edinburgh, UK between August 2015 and March 2020. Patients had to be diagnosed with clinically significant bronchiectasis (regular cough and sputum production with increased risk of chest infections), with radiological confirmation using an HRCT chest scan. Patients included in the study, were those with a complete dataset including serum IgG subclasses levels. Patients that had IgG deficiency, IgG1 deficiency, IgG3 deficiency, IgG4 deficiency, IgA deficiency and IgM deficiency were excluded.

### Data and variables

All patient information was collected from electronic patient records. Bronchiectasis severity index (BSI) score was calculated at the time of enrolment and 1 year after this. A BSI score of 1–4 is considered mild bronchiectasis; 5–8, moderate bronchiectasis and 9 or more, severe bronchiectasis.^5^^,^^9^

### Statistics

In the multivariable model, the following were included (Table 1): serum IgG2 levels; lung function (FEV_1_% predicted and FEV_1_/FVC); body mass index; MRC breathlessness score; age; sex; number of bronchiectatic lobes; bacterial colonization; co-existent asthma; co-existent COPD; rhinosinusitis; allergic bronchopulmonary aspergillosis; primary ciliary dyskinesia; past pneumonia; past Tuberculosis (TB); gastroesophageal reflux disease; ischaemic heart disease; rheumatoid arthritis; other inflammatory arthritis or auto-immune disease (systemic lupus erythematosus, auto-immune haemolytic anaemia, Sjogren’s syndrome, ankylosing spondylitis); the use of long-term immunosuppressant drugs (prednisolone ≥10 mg/day for ≥28 days, mycophenolate mofetil, hydroxychloroquine, methotrexate and azathioprine for ≥ 28 days) and long-term antibiotics for ≥28 days. For the multivariable model, those features were classified as 0, 1, 2 and 3, according to individual parameters; as detailed in Table 1. Response variables (number of bronchiectasis exacerbations and need for hospitalization in a 2-year period before this study) were dichotomized to ‘0’ = <3 exacerbations and ‘1’ = ≥3 exacerbations or ‘0’ = no hospitalization and ‘1’ = had hospitalization, respectively.

**Table 1.** Variables used in the study

	0	1	2	3
Exacerbation	<3	≥3		
Hospitalization	No	Yes		
IgG2	>4.45g/l	3.54–4.45 g/l	2.68–3.53 g/l	<2.68 g/l
FEV_1_%	≥80%	50–80%	30–49%	<30%
FEV_1_/FVC	≥80%	<80%		
Lobe	<3	≥3		
Sex	Male	Female		
Smoking	No	Yes		
Age	<50	50–69	70–79	>79
BMI	18.5–25	26–30	>30	<18.5
MRC	1,2,3	4,5		
Bacterial colonization	No	Yes		
Asthma	No	Yes		
COPD	No	Yes		
Rhinosinusitis	No	Yes		
Past pneumonia	No	Yes		
Past TB	No	Yes		
ABPA	No	Yes, no steroids	Yes, with steroids	
Primary ciliary dyskinesia	No	Yes		
Gastroesophageal reflux disease	No	Yes		
Ischaemic heart disease	No	Yes		
Cerebrovascular disease	No	Yes		
Rheumatoid arthritis	No	Yes		
Other inflammatory arthritis or auto-immune disease	No	Yes		
Long-term antibiotics	No	Yes		
Long-term immunosuppressant drugs	No	Yes		

A binary logistic regression analysis using a backward model was conducted; *P-values* < 0.05 was considered significant. Odds Ratios (OR) and 95% confidence intervals (CI) were calculated. The Hosmer–Lemeshow test was used to determine the suitability of the model, with *P* > 0.05 demonstrating that the model fit the data. For the 1-year longitudinal analysis, the comparison change of BSI scores between different IgG2 levels in patients were analysed using one-way ANOVA and Mann–Whitney U-test. SPSS version 25.0 was used, carrying out a binary logistic regression using a backward model for the multivariable model. Prism version 8.0 was used for the one-way ANOVA and Mann–Whitney U-test.

### Ethics approval

This was a retrospective study and NHS Lothian has given Caldicott approval, reference number CG/DF/20113.

## Results

There were 674 individual bronchiectasis patients fit the criteria were included from August 2015 to March 2020 (Table 2). Among these patients, 34.7% had co-existent asthma and 11.2% had co-existent COPD. Of about 4.3% were on long-term oral or inhaled antibiotic therapy (inhaled gentamicin and colomycin, oral erythromycin, clarithromycin, azithromycin, co-trimoxazole, penicillin and amoxicillin with clavulanic acid). There were 8% taking long-term immune suppressant drugs (3.2% on Prednisolone ≥10 mg/day for ≥28 days, 0.8% on hydroxychloroquine, 1.8% on methotrexate, 0.4% on mycophenolate and 0.3% on azathioprine).

**Table 2.** Patients information (*n* = 674)

Gender (F/M)	410/264
Current smokers	66 (8.4%）
Ex-smoker	282 (36%)
Pack year	21.6 (6–31)
Exacerbation	3 (1–4)
MRC breathlessness score	1.2 (1–1)
Number bronchiectasis lobes	2.5 (1–3)
Age	63 (57–74)
BMI, kg/m^2^	26.7 (12.5–29.7)
FEV_1_% predicted	79% (63–96%)
FVC% predicted	95% (80–109%)
FEV_1_/FVC	68.3% (60.6–78.1%)
BSI score	6.3 (4–8)
Hospitalization	101 (12.9%)
Asthma	272 (34.7%)
COPD	88 (11.2%)
Rhinosinusitis	31 (4.0%)
ABPA	34 (4.3%)
Past pneumonia	96 (12.3%)
Past TB	69 (8.8%)
Primary ciliary dyskinesia	4 (0.5%)
Gastroesophageal reflux disease	37 (4.7%)
Rheumatoid arthritis	29 (3.7%)
Other arthritis and auto-immune diseases	16 (2%)
Long-term immune suppressant drugs	59 (8%）
Long-term antibiotics	34 (4.3%）

Data presented as median interquartile range or *n* (%).

BMI, body mass index; FEV1% predicted, forced expired volume in 1 s as a percent predicted; FVC% predicted, forced vital capacity as a percent predicted; BSI, Bronchiectasis severity index.

Independent risk factors for three or more exacerbations per year.

Patients’ serum IgG2 levels were categorized into four groups according to quartiles: <2.68 g/l, 2.68–3.53 g/l, 3.54–4.45 g/l and >4.45 g/l. Independent risk factors for three or more exacerbations per year were: IgG2 <2.68 g/l, IgG2 levels 2.68–3.53 g/l and 3.54–4.45 g/l; hospital admission in the preceding 2 years; bacterial colonization with potential pathogenic organisms in the past 12 months and asthma (Table 3).

**Table 3.** Results of binary logistic regression models using bronchiectasis exacerbation (three or more per year) as the dependent variable

Variables in the equation								
	B	S.E.	Wald	d*f*	Sig.	OR	95% CI	
							Lower	Upper
IgG2 (3.54–4.45 g/l)	0.647	0.267	5.882	1	0.015**	1.909	1.132	3.219
IgG2 (2.68–3.53 g/l)	0.584	0.255	5.268	1	0.022*	1.793	1.089	2.954
IgG2 (<2.68 g/l)	0.863	0.278	9.676	1	0.002**	2.371	1.376	4.085
Hospital admission	0.677	0.289	5.485	1	0.019**	1.967	1.117	3.465
Bacterial colonization	0.611	0.189	10.510	1	0.001***	1.843	1.273	2.666
Asthma	0.663	0.200	11.017	1	0.001***	1.940	1.312	2.870

* P ≤ 0.05; ** P ≤ 0.01; *** P ≤ 0.001.

### Independent risk factors for hospital admissions

Independent risk factors for hospital admissions were: forced expired volume in 1s <50% predicted; colonization with *Pseudomonas aeruginosa* in the past 12 months, past pneumonia, three or more exacerbations per year and being on long-term antibiotics (Table 4).

**Table 4.** Results of binary logistic regression models using hospitalization as the dependent variable

Variables in the equation								
	*B*	SE	Wald	d*f*	Sig.	OR	95% CI	
							Lower	Upper
FEV_1_%P (30–49%)	0.808	0.407	3.943	1	0.047*	2.243	1.011	4.980
FEV_1_%P (<30%)	1.545	0.624	6.139	1	0.013*	4.688	1.381	15.910
*P.aeruniosa* colonization	0.806	0.398	4.105	1	0.043*	2.239	1.027	4.885
Past pneumonia	0.865	0.396	4.769	1	0.029*	2.376	1.093	5.165
Long-term antibiotic	1.260	0.490	6.629	1	0.010**	3.527	1.351	9.205
Exacerbation	0.739	0.286	6.705	1	0.010**	2.095	1.197	3.666

Impact of IgG2 levels on longitudinal changes of BSI score.

**Figure 1.**
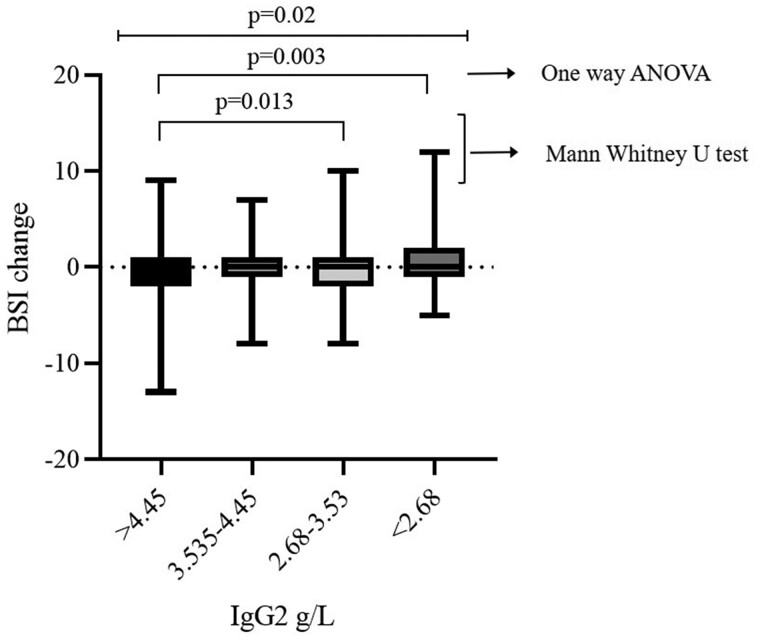
The change of bronchiectasis BSI score in 1 year by serum IgG levels. Data are showed in box and whisker plots (min to max). *P*-values were calculated by one-way ANOVA and Mann–Whitney U-test.

The longitudinal impact of IgG2 levels on the severity of bronchiectasis over 1 year was explored. Patients with lower IgG2 (≤3.53 g/l) had increased progression (increased bronchiectasis severity score) compared with patients with high IgG2 levels (>4.45 g/l) (*P* = 0.013 and 0.003) (Figure 1).

## Discussion

There are a number of factors that independently predict exacerbation frequency in bronchiectasis, including gastro-oesophageal reflux, comorbid cardiovascular disease, co-existent asthma, age, duration of symptoms, FEV_1_ ≤50% predicted, ≥3 bronchiectatic lobes and airway colonization with *P. aeruginosa.*^19–22^ Research has also demonstrated that COPD, sputum neutrophil elastase activity, previous viral infection and high airway bacterial loads are associated with more exacerbations in bronchiectasis.^23–26^ Age, heart failure, previous hospitalization due to bronchiectasis, use of proton pumps inhibitors and high BSI scores (FACED and BSI) are associated factors for hospital admission.^10^^,^^27^

In this study, the authors picked the threshold of three or more exacerbations, as this is the trigger to augment treatment in the BTS and ERS guidelines.^6^^,^^28^ IgG2 <2.68 g/l, IgG2 levels 2.68–3.53 g/l and 3.54–4.45 g/l, prior hospitalization in the preceding 2 years, bacterial colonization and underlying asthma were independent factors for ≥3 exacerbations per year. Bacterial colonization was defined as the isolation of potentially pathogenic organisms from lower respiratory tract samples on two or more occasions during the preceding year when the patient was in the stable state. All these risk factors make intuitive sense and are in keeping with the above literature. Due to immune response to chronic infections, IgG levels in bronchiectasis patients are higher than those in unaffected individuals.^11^^,^^29^ It is interesting that even levels of 3.54–4.45 g/l were associated with increased infections.

In this study, for hospitalization in the preceding 2 years, FEV_1_ <50% predicted, colonization with *P. aeruginosa* in the past 12 months, past pneumonia, three or more exacerbations per year and being on long-term antibiotics were independent factors for hospitalization. These risk factors are both biologically plausible and consistent with other publications in this field, revealing that independent factors related to severe disease with recurrent exacerbations, impaired lung function and needing a long-term antibiotic.

IgG2 subclass deficiency was not uncommon in our bronchiectasis cohort. Among the 674 patients, isolated IgG2 deficiency was identified in 24.5% in this study, defined as being below the lower limit of the reference range from the NHS laboratory.

Patients in the lowest quartile of IgG2 had more than two times the rate of exacerbations and hospitalizations. Furthermore, lower IgG2 levels were associated with faster progression of bronchiectasis severity, as demonstrated by a greater increase in BSI score over 1 year compared.

As a complement to the results from multivariate analysis, we also compared the exacerbation and hospital frequency between patients with IgG2 level <2.68 g/l and patients with IgG2 level more than 4.45 g/l using Mann–Whitney U-test, patients had low IgG2 levels had significantly more frequent exacerbations and hospitalization frequency (*P* = 0.003 and *P* = 0.01) (Figure 1). Although low IgG2 level was not a risk factor for hospital admission, the patients with low IgG2 had significant more frequent hospital admissions than those patients with higher IgG2 levels.

There are limitations of this study. First, this is a single-centre study. Secondly, there no data were available on IgG pneumococcal vaccine responses. However, there is a full dataset with accurate data collection on number of exacerbations, rate of hospitalization and immunoglobulins and their subclasses. All patients have regular review in the bronchiectasis clinic 6–12 monthly and keep a diary card of exacerbations, and at each consultation, the frequency of exacerbations and hospitalization is recorded. Also, this study used multivariate analysis and has longitudinal BSI follow-up.

The BTS guidelines recommend checking IgG, IgA and IgM but not to routinely check IgG subclasses.^6^ Further studies are needed to explore whether similar data are seen in other centres in the UK and internationally. Immunoglobulin prophylaxis can significantly reduce recurrent respiratory tract infections in IgG subclass deficiency^25^ and intravenous immunoglobulin therapy significantly reduced exacerbation frequency in COPD patients with hypogammaglobulinemia.^30^ These treatments may be beneficial for IgG2 deficient patients with bronchiectasis, but no study to date has yet explored the effect of immunoglobulin therapy on IgG2 deficient patients with bronchiectasis.

## Interpretation

Reduced IgG2 levels were an independent predictor for bronchiectasis exacerbations and have increased disease progression. Further exploratory studies are needed.

## Take home message

Reduced IgG2 levels were an independent predictor for bronchiectasis exacerbations and have increased disease progression. Further exploratory studies are needed.

## Author contributions

Y.Z. contributed to study design, collected and interpreted the data and wrote the manuscript. A.C., K.H.R, S.D., J.C. and K.C. contributed to collecting patients’ information. A.G.R contributed to the study design. A.T.H. contributed to study design, interpretation of data and writing of the manuscript.

## Conflict of interest

None declared.
